# Can weight loss prevent cancer?

**DOI:** 10.1038/sj.bjc.6604623

**Published:** 2008-08-26

**Authors:** K Y Wolin, G A Colditz

**Affiliations:** 1 Department of Surgery and Alvin J Siteman Cancer Center, Washington University School of Medicine, St Louis, MO, USA

**Keywords:** weight gain, obesity, weight loss, prevention

## Abstract

We review and update evidence on obesity, weight gain and weight loss in relation to leading cancers since the International Agency for Research on Cancer report of 2002. Emphasis is placed on the time course of disease and implications for weight control to prevent cancer. We conclude that weight loss could prevent a major portion of common cancers.

In 2002, the International Agency for Research on Cancer (IARC) reviewed the evidence for an association between body weight and cancer (International Agency for Research on Cancer, 2002). The IARC concluded that sufficient evidence existed for avoiding weight gain to protect against cancer. This evidence applied to colon, breast (postmenopausal), endometrial, kidney (renal cell) and oesophageal cancers. No effect was found for premenopausal breast cancer, and insufficient evidence was available for all other cancers. The IARC further found insufficient evidence for a protective benefit of intentional weight loss for any cancer site.

The cancers for which insufficient evidence was found include ovarian, prostate, lung, pancreatic, head and neck, testicular, thyroid, gall bladder, melanoma and cervical cancer. The IARC committee did not consider evidence for non-Hodgkin's lymphoma, myeloma or meningioma as very few studies could be found to adequately evaluate risk.

In 2007, the World Cancer Research Fund and American Institute for Cancer Research (WCRF/AICR) issued summary findings on the association of obesity with cancer (World Cancer Research Fund/American Institute for Cancer Research, 2007). Convincing evidence was found for body fatness and colorectal, pancreatic, oesophageal, postmenopausal breast, kidney and endometrial cancers as well as for the association of abdominal fatness and colorectal cancer. Probable evidence existed for body fatness and gall bladder and premenopausal breast cancers and for abdominal fatness and endometrial, pancreatic and postmenopausal breast cancers. Suggestive evidence was found for body fatness and lung and liver cancers. Despite the increasing wealth of data on obesity and cancer, WCRF/AICR found sufficient evidence to review the association with weight gain only for postmenopausal breast cancer, concluding that a ‘probable’ association existed.

Thus, despite the extensive evidence showing a deleterious effect of overweight and obesity on cancers (except for premenopausal breast cancer where obesity appears protective), relatively little data exist on the effects of weight gain or weight loss on the risk of cancer. The lack of data on weight loss is most likely a function of the small number of individuals able to achieve sustained weight loss.

## Methods

We review the evidence presented in the IARC and WCRF/AICR reports and studies published subsequently for several major cancers. A comprehensive review of studies published and indexed on PubMed since the two reports was carried out. Selected studies that highlight key findings are presented.

## Results

### Colon cancer

Colon cancer is the second leading cause of cancer-related deaths in the United States and the United Kingdom (http://info.cancerresearchuk.org/cancerstats/mortality/cancerdeaths/) and the fourth leading cause of cancer-related deaths worldwide (World Health Organization, 2008). The risk of developing colon cancer is thought to be reduced through a number of lifestyle factors, with maintaining a healthy weight and physical activity being among the most important. The IARC report found consistent positive associations between body fatness, as indicated by body mass index (BMI), and risk of colorectal cancer. Risk of colon cancer was increased by 50–100% when comparing the highest and lowest categories of BMI with a linear trend across levels of BMI. WCRF/AICR concurred in 2007, finding convincing evidence that body fatness increased the risk of colorectal cancer. Two subsequent meta-analyses also found a significantly increased risk of colon cancer with increasing BMI (Dai *et al*, 2007; Larsson and Wolk, 2007). Evidence for rectal cancer is less certain, with some studies finding weak positive associations (Dai *et al*, 2007; Larsson and Wolk, 2007) and others finding little evidence for an association (International Agency for Research on Cancer, 2002).

Overweight and obesity appear to be more strongly related to colon cancer incidence in men than in women (International Agency for Research on Cancer, 2002; Dai *et al*, 2007; Larsson and Wolk, 2007). There is some evidence that hormones may modify the association of obesity with colon cancer in women, as oestrogen may increase susceptibility to the carcinogenic effects of higher insulin levels associated with obesity (Giovannucci, 2002). The IARC report also found consistently increased risk of colorectal adenomas when comparing the highest to lowest BMI strata, which was supported in subsequent research (Kim *et al*, 2007). Gender differences are smaller for colorectal adenoma than for colorectal cancer.

In addition to BMI, evidence exists that central body fat distribution and abdominal fatness are associated with increased risk of colon cancer and adenoma (International Agency for Research on Cancer, 2002; Engeland *et al*, 2005; World Cancer Research Fund/American Institute for Cancer Research, 2007). Gender differences were also found for abdominal fatness (Larsson and Wolk, 2007). Recent meta-analyses have provided further support for the association (Dai *et al*, 2007; Larsson and Wolk, 2007).

Limited evidence is available on the association between weight change and the risk of colon cancer. Studies suggest that adult weight gain increases the risk of colorectal adenoma, the precursor to most colorectal cancers (Bird *et al*, 1998; Kono *et al*, 1999). For example, in a study of Japanese men undergoing colonoscopy, Kono *et al* (1999) found that current, but not past, BMI was positively associated with adenoma risk and that weight gain over the past 10 years was positively associated with risk. Researchers extracted weight for the previous 10 years from medical records where it was recorded during the men's biannual health exam. Men who gained 6 kg or more were twice as likely to have an adenoma as men who lost 2 kg or more. The associations in these studies were strongest for larger adenomas. Furthermore, Kono *et al* found that weight gain combined with a high waist to hip ratio significantly increased risk, suggesting that the distribution of weight gain may be as important as the gain itself.

The IARC found limited evidence for an association between weight change and the risk of colorectal cancer (International Agency for Research on Cancer, 2002). Cohort studies examined weight change from early adulthood to later in life and found modest increases in risk (RRs between 1.4 and 1.6). Case–control studies provided additional support. More recent evidence indicates that weight gain in adulthood appears to increase the risk of colon cancer. In a case–control study in Canada, men who gained 21 kg or more since the age of 20 years were at 60% increased risk of colorectal cancer as compared to men who had gained 1–5 kg (Campbell *et al*, 2007). The association was stronger when rectal tumours were excluded, suggesting that studies that examine the association of weight gain on colorectal cancer may underestimate the association for colon cancer. No association between weight gain and colorectal cancer risk was observed among women. Another study of men and women found that compared to those who had remained BMI stable, those who increased their BMI from the age of 30 or 50 years to diagnosis were at 25–35% lower risk of colorectal cancer (Russo *et al*, 1998). Finally, a study of Austrian adults found evidence for an inverse association between weight loss and colon cancer in men (Rapp *et al*, 2008).

Thus, weight appears to influence colon cancer risk at multiple stages of carcinogenesis including initiation, promotion and progression. Given the association with attained weight as a dominant risk factor, avoiding weight gain could help avoid risk. Because of the link between obesity and both large adenomas and colon cancer, obesity may be acting at a later stage of tumour development. Several mechanisms have been proposed to explain the link between obesity and colon cancer (Bray, 2002; Giovannucci, 2002). Weight loss and increased activity reduce the circulating levels of insulin, a growth factor for colonic epithelial cells, and improve insulin sensitivity. Weight loss may also affect IGF and IGFBP levels. Insulin and IGF stimulate proliferation, inhibit apoptosis and have effects on cellular differentiation and angiogenesis. Weight gain may also act by increasing levels of prostaglandins, which decrease intestinal motility and increase cell proliferation. Additional research on the timing of weight loss and how it affects these potential mechanisms is merited.

### Breast cancer

Breast cancer is a major cause of morbidity and the leading cause of cancer mortality among women globally accounting for over 500 000 deaths per year (World Health Organization, 2008). The IARC report concluded that obesity and weight gain are directly and positively related to postmenopausal breast cancer (International Agency for Research on Cancer, 2002). The WCRF/AICR concurred, finding convincing evidence for body fatness and probable evidence for abdominal fat and weight gain (World Cancer Research Fund/American Institute for Cancer Research, 2007). A meta-analysis conducted as part of the WCRF/AICR report found a 5% increase in risk of postmenopausal breast cancer with each 5 kg of weight gain.

Because sustained weight loss is not common, epidemiologic data have been sparse on the benefits of weight loss in relation to reduced risk of breast cancer. Weight gain was associated with increased risk of postmenopausal breast cancer in several cohort studies, particularly among women not taking supplemental hormones (Eliassen *et al*, 2006; Han *et al*, 2006; Ahn *et al*, 2007). In the prospective Nurses' Health Study, weight gain after menopause was directly related to risk of breast cancer (Eliassen *et al*, 2006). As compared to women with stable weight, weight loss significantly reduces risk. On the basis of these data, it is estimated that adult weight gain accounts for 24% of postmenopausal breast cancer and that weight gain after menopause accounts for 7% of breast cancer among women who do not use postmenopausal hormone therapy. However, these findings may not translate to all groups, as no association was found among African-American women, (Palmer *et al*, 2007), though the reasons for this remain unclear.

Recent studies have elucidated mechanisms underlying weight, weight loss and reduced risk of postmenopausal breast cancer. Endogenous hormones are related not only to adiposity in postmenopausal women but also to a oestrogen receptor-positive breast cancer (Missmer *et al*, 2004). A year-long randomised trial of activity among postmenopausal women shows that among women who lost body fat, serum oestrogen levels were decreased (McTiernan *et al*, 2004).

In contrast to the findings for postmenopausal breast cancer, IARC found limited but supportive data for an inverse association between both BMI and weight gain with premenopausal breast cancer. WCRF/AICR concluded that there was a probable relation between body fatness and premenopausal breast cancer, but it did not examine weight gain or abdominal fatness. The IARC found no support for a relation between premenopausal breast cancer and central adiposity.

### Prostate cancer

Prostate cancer is the sixth leading cause of cancer death among men worldwide (World Health Organization, 2008), the second leading cause of cancer death among US and British (http://info.cancerresearchuk.org/cancerstats/mortality/cancerdeaths/) men and the most common type of cancer found in men in the United States and the United Kingdom (http://info.cancerresearchuk.org/cancerstats/incidence/males/). Although 1 in 6 US men will get prostate cancer in his lifetime, only 1 in 35 will die from the disease. Links between prostate cancer and lifestyle factors are less clear than those for breast and colon cancer.

In its 2002 review, IARC found insufficient evidence to draw a conclusion about the relationship between obesity and the risk of prostate cancer (International Agency for Research on Cancer, 2002). However, there was a suggestion what whereas obesity was not associated with the risk of prostate cancer overall, it might be associated with fatal or aggressive disease. There were also inconsistent associations between weight in young adulthood and risk of prostate cancer. A subsequent meta-analysis found a weak but statistically significant positive association between BMI and prostate cancer risk, with stronger associations for more advanced disease (MacInnis and English, 2006). In the 2007 WCRF/AICR report, insufficient evidence was found for body and abdominal fatness (World Cancer Research Fund/American Institute for Cancer Research, 2007).

Recent evidence suggests that obesity increases the risk for advanced prostate cancer and prostate cancer mortality, but not the risk of less aggressive disease. Several studies provide significant support for the hypothesis that obesity increases the risk of aggressive prostate cancer (Freedland and Platz, 2007; Rodriguez *et al*, 2007; Wright *et al*, 2007).

Only a handful of studies have specifically examined the effects of weight gain or loss on the risk of prostate cancer. For example, in the Cancer Prevention Study II Nutrition Cohort, Rodriguez *et al* observed a significantly decreased risk of high-grade prostate cancer in men who lost >11 lbs as compared to those who were weight-stable (change of ⩽5 lbs) (Rodriguez *et al*, 2007). Similarly, Cerhan observed a significant trend of reduced risk with weight loss from the age of 50 years and increased risk with weight gain from the age of 50 years relative to no change (Cerhan *et al*, 1997). The association for BMI change from the age of 50 years was stronger among men with BMI >24.4 kg m^−2^ at the age of 50 years. Men who gained >10% weight and had a BMI >24.4 kg m^−2^ were twice as likely to develop prostate cancer as men with BMI ⩽24.4 kg m^−2^ who, at baseline, were within 5% of their BMI at age 50. Finally, Wright *et al* found that weight gain from the age of 18 years significantly increased the risk of fatal, but not incident, prostate cancer (Wright *et al*, 2007). However, not all studies have found an association (Schuurman *et al*, 2000; Song *et al*, 2008).

A number of potential mechanisms have been hypothesised to explain the observed associations and variations in study findings (Freedland and Platz, 2007). Obesity may influence prostate cancer detection. Obese men may have lower PSA levels, though not all data support this. Obesity also decreases SHBG levels, which are inversely associated with prostate cancer risk. Finally, evidence indicates that obese men have larger prostates, which may impair the ability of biopsy to detect tumours, as biopsies sample a smaller fraction of the total prostate.

### Oesophageal cancer

The IARC found sufficient evidence and WCRF/AICR found convincing evidence that body fatness increases the risk of oesophageal cancer. Specifically, obesity increases the risk of oesophageal adenocarcinoma (but not squamous cell carcinoma). Evidence for weight loss reducing risk of oesophageal reflux and hence the risk of adenocarcinoma of the oesophagus is growing (Jacobson *et al*, 2006). Merry *et al* examined the change in BMI and found that a gain of 1 kg m^−2^ after the age of 20 years increased the risk of oesophageal adenocarcinoma by 14%; those with a gain greater than or equal to 8 kg m^−1^ had 3.4 times the risk as those with an increase between 0 and 3.9 kg m^−2^ (Merry *et al*, 2007). A significant increase in risk of gastric cardia adenocarcinoma was also found. Subsequent reports have also found increased risk for abdominal fatness, body fatness and oesophageal adenocarcinoma (Kubo and Corley, 2006; Corley *et al*, 2008). Higher BMI was also associated with gastric cardia adenocarcinomas (Kubo and Corley, 2006), but abdominal fatness was not (Corley *et al*, 2008).

### Pancreatic cancer

Although IARC found insufficient evidence in 2002, by 2007 the WCRF/AICR had found convincing evidence that body fatness increased risk of pancreatic cancer and abdominal fatness probably increased risk. In a meta-analysis of cohort data, the WCRF/AICR reported a 14% increased the risk of pancreatic cancer for every 5 kg m^−2^ increase in BMI. Subsequent reports have been conflicting (Lo *et al*, 2007; Stolzenberg-Solomon *et al*, 2008).

### Endometrial cancer

In 2002, IARC found sufficient evidence that obesity increased the risk of endometrial cancer. The WCRF/AICR meta-analysis found a 52% increase in risk for every 5 kg m^−2^ increase in BMI and a 31% increase in risk for every 5 kg m^−2^ as a young adult. Although this does not provide direct evidence that weight gain increases the risk of endometrial cancer, it does suggest that changes in BMI from young adulthood are important. The WCRF/AICR also found probable evidence that abdominal fatness increases the risk of endometrial cancer.

### Kidney and renal cell cancer

Nearly all studies included in the WCRF/AICR report found an increased risk of kidney cancer with increased BMI. A meta-analysis found a 31% increase in risk for each 5 kg m^−2^ increase in BMI based on cohort data and a 205% increase in risk based on case–control data. More recent reports have supported the WCRF/AICR findings and have suggested that weight cycling may increase the risk of renal cell cancer (Luo *et al*, 2007; Setiawan *et al*, 2007).

## Summary of evidence

Studies now support weight loss after menopause as a means to reduce risk of breast cancer. Weight loss is associated with lower risk of gastroesophageal reflux, a plausible component of the pathway from adult obesity to adenocarcinoma of the oesophagus. Limited evidence also suggests that weight loss may reduce the risk of prostate cancer. For other cancer sites, there is limited evidence that weight gain and BMI are related to cancers. Obviously, to increase adult BMI, one must gain weight, and accordingly the IARC concluded its recommendation to avoid adult weight gain. Thus, at a minimum, recommendations should emphasise weight maintenance. Given the substantial body of evidence from randomised controlled trials that weight loss reduces the risk of diabetes (Diabetes Prevention Program Research Group, 2002) and improves blood pressure and blood lipids (NHLBI Obesity Education Initiative Expert panel on the Identification, 1998), weight loss can be recommended to produce health benefits for overweight and obese adults. We outline strategies for weight loss in an accompanying Supplementary Information.

## Why a prevention trial with cancer as an end point is not feasible

The challenges of sustaining preventive interventions over the long time periods necessary for primary prevention of cancer have been thoroughly described by Zelen (1988). Importantly, he emphasised that over time non-compliance would reduce the power of a study as the intervention and control arms become more similar. This was observed empirically in the Women's Health Initiative randomised controlled trial where over 42% of women assigned to combination oestrogen plus progestin had ceased taking their medication during an average of 5.2 years on study and some 10.7% of women assigned to placebo had begun taking postmenopausal hormone therapy (Rossouw *et al*, 2002). In the study's diet intervention, adherence was low (31.4% at year 1) and fell to 14.4% at year 6 (Beresford *et al*, 2006). The Women's Health Initiative Dietary Modification Trial assigned women to a dietary modification that resulted in a weight loss among the intervention group, whereas the control group gained weight between baseline and year 3 (Beresford *et al*, 2006). The trial found a nonsignificant reduction in the risk of colorectal or breast cancers (Prentice *et al*, 2007). There was a suggestion of decreased risk of ovarian cancer (Prentice *et al*, 2007). However, it is unclear whether the risk reduction was due to the low fat diet or the concurrent weight loss.

A common approach in cancer prevention trials has been to identify participants at high risk of cancer. For weight loss and cancer prevention, this translates to recruiting overweight and obese participants, a strategy that can very likely lead to substantial ethical concerns regarding stopping rules based on prevention of diabetes and other short-term health benefits likely to be observed long before cancer risk reduction is detected.

In addition, for colon cancer, screening strategies in usual care are likely to eliminate detectable benefits or reduce the incidence of disease and thereby lead to large sample size necessary to detect differences in outcomes in a prevention trial. Likewise for breast cancer, the growing use of Selective Oestrogen Receptor Modulators among high-risk women will most likely reduce the effect of weight loss and associated reductions in hormone levels.

On the basis of these concerns, it is unlikely that in the near future a cancer prevention trial will focus on weight loss as a primary prevention strategy, despite the evidence for benefit through weight control and weight loss that we have reviewed.

## Conclusion

If individuals achieve and maintain weight loss, we could prevent substantial cancer burden. This is most evident for postmenopausal breast cancer. The time frame for the benefits of reduced cancer incidence after successful weight loss remains unclear for most cancers.
