# Supplementary material for: Can weight loss prevent cancer?
Source: Br J Cancer. 2008 Aug 26;99(7):995–9. doi: 10.1038/sj.bjc.6604623 (PMC2567071; doi:10.1038/sj.bjc.6604623)
Supplement: Supplementary Information [file 6604623x1.doc]

**Overview of strategies to promote weight loss**

In the accompanying manuscript, we outline the evidence that maintaining a healthy weight reduces the risk of cancer. Given the prevalence of overweight and obesity, weight loss is a sound strategy for reducing risk of cancer. Here we briefly outline strategies to maintain weight and achieve sustained weight loss.

# *Strategies to maintain weight and achieve weight loss*

Strategies to achieve weight loss aim to alter one or more of the many factors that influence energy balance. Using the ecological model of health behavior, Sallis and Owens (Sallis & Owen, 1999) have categorized these factors as follows:

- Intrapersonal factors: Factors that occur within the individual’s mind or body, including biological, cognitive, and behavioral factors.
- Social environment factors: Social and cultural factors that promote or discourage a given behavior.
- Physical environment factors: Factors within the natural environment (such as weather or geography) or constructed environment (such as the transportation system or infrastructure for recreation) that promote or discourage a given behavior.

Lack of energy expenditure in the form of physical activity is a leading determinant of higher body mass. For those who are already overweight, physical activity, in combination with dietary changes, is important for achieving, and particularly for maintaining, weight loss. There is increasing evidence (Benne*tt et* al, 2006a) that attaining in excess of 9000 daily steps as an indicator of physical activity, is associated with a lower likelihood of being obese, including among lower income and racial/ethnic minority populations. Television (TV) viewing is a highly prevalent sedentary activity; Americans watch at least 4 hours of TV each day and TV viewing is the most prevalent activity after work and sleep. TV viewing is positively associated with excess body weight among children and adults (Berk*ey et* al, 2003; *Hu et* al, 2003). TV viewing may also promote consumption of calorically dense foods through: 1) advertising and other media content, and; 2) displacement of time that could be spent on activities that burn calories (Benne*tt et* al, 2006b). The strong and consistent association between TV viewing and obesity suggests the importance of including reduction in TV hours as a target in behavioral weight reduction efforts.

The NHLBI evidence review on weight loss evaluated 86 RCTs and concluded that “low calorie diets are recommended for weight loss in overweight and obese persons” (evidence category A) and that “Physical activity is recommended as part of a comprehensive weight loss therapy and weight control program because it modestly contributes to weight loss” (evidence category A)(NHLBI Obesity Education Initiative Expert panel on the Identification, 1998). Specific recommended intake levels vary based on a number of factors including current weight, activity levels, and weight loss goals. There are many other behavioral factors that influence weight loss and may be more effectively intervened upon over an extended intervention period to achieve sustained weight loss. We outline several of these in Figure 1. Rather than focus on specific calorie calculations, we recommend behavioral targets that should lead to an energy deficit as well as increase and maintain patient motivation.

## Health Care Settings

The Diabetes Prevention Trial demonstrates the efficacy of an intensive health care provider-based intervention to reduce weight and increase physical activity over a 24 month period and thereby prevent diabetes (Diabetes Prevention Program Research Group, 2002). Translating this efficacy study to approaches that work in the broader clinical setting remains to be demonstrated as effective. One approach, derived from methodology originally developed by the National Cancer Institute to guide physicians in counseling their patients to quit smoking. The “Five A’s” model guides clinicians through a counseling session, with each “A” corresponding to a brief behavioral intervention—assess, advise, agree, assist, arrange — which together have been shown effective.

Interactive technologies can also address a wide variety of health behavior domains simultaneously. Thus, interactive technology can provide a streamlined, consistent method for conducting many aspects of evidence-based behavior change counseling, including assessing current health behaviors, identifying barriers to change, and allowing the patient to set goals and select relevant activities, and arranging follow-up support.

Evidence from randomized trials demonstrates that use of interactive technologies supports lifestyle behavior change (Glasg*ow et* al, 2004), although most of this research has been conducted using a single-disease and/or single behavior change focus. Practice-based research indicates that clinicians who have limited financial, space, and personnel resources favor tools that address co-morbid conditions and health behaviors simultaneously. In addition, tools that can be accessed via patient’s homes or from community settings are appealing to clinicians whose barriers to providing self-management counseling and follow-up are great.

Increasingly, Web-based tools for supporting positive lifestyle changes are making their way into the clinical setting. While it’s currently unclear how effective such interventions are, an increasing number of studies are beginning to assess their potential, both in the health care setting and beyond.

B. The Social Environment in Health Behavior Change

The impact of social-ecological factors on health is a key tenet of public health. Multi-level models that emphasize interactive systems typically consider the individual’s immediate environment (e.g. family or neighborhood), and other settings that are important to daily life (e.g. workplace or primary care). Although the data on social/ environmental influences on health behaviors and diabetes are compelling and growing, broader applications are still needed.

## C. Environment and Policy

Environment and policy approaches to increasing physical activity have the potential to influence large segments of the population simultaneously and, therefore, can often be less costly (on a per capita basis) and more enduring than approaches that focus on convincing people to be more active. Rather than persuade individuals to change, environmental and policy approaches aim to make it easier for individuals to choose and maintain physically active lifestyles. They do this by changing social norms (for example, making it more acceptable to commute to work by bicycle or foot or to exercise during lunch hour) or by changing the environment (for example, improving road and sidewalk conditions for pedestrians and cyclists or making workplace stairways as safe, well-lit, and accessible as elevators).

Given the inherent difficulties of evaluating a population-based approach to behavior change, there is little published data on the effectiveness of environment and policy approaches to increasing physical activity. In a 1998 review of the topic, Sallis et al. found only seven published studies, with interventions ranging in complexity from the posting of simple signs encouraging the use of stairs to the opening of 14 publicly-funded recreation centers in Belfast (Sall*is et* al, 1998). Methodological flaws in these studies eventually precluded the authors from reaching firm conclusions about their efficacy.

D. Summary of Strategies

Because the factors that influence energy balance range from the individual to the environment, efforts to shift the population distribution of activity upward and diet to healthier choices will require emphasis on all of the intervention points mentioned above: health care settings, communities, and environment. The challenge for prevention is to develop a comprehensive approach to physical activity and weight control that targets individual and environmental barriers simultaneously.

#### Safety Issues

Although the benefits of weight maintenance and greater physical activity are well established for many chronic diseases, the risks must also be considered. These include injuries, alteration in glucose control for diabetics, and sustained increases in joint pain. In general, these risks are outweighed by the vast benefits of weight loss and increased physical activity and can generally be reduced with simple steps.

**Diet, physical activity, and behavioral goals for sustained weight loss and avoid weight gain.**

### Diet Goals

1. Eliminate consumption of sugar sweetened beverages (fizzy drinks). Choose from alternatives including:
   - 1. Water
     2. Tea
     3. Diet soda (fizzy drinks)
2. Eat a breakfast each day. Examples of a healthy breakfast include:
   1. ¾ cup high fiber cold cereal such as bran flakes, fiber one, or kashi with at least 5 grams of fiber or more. Serve with ¾ cup 1% or skim milk and ½ banana. (see skills building section on how to read a food label)
   2. 1 cup cottage cheese with 1 cup fresh fruit
   3. 1 cup cooked oatmeal and 1 piece of fruit
   4. 2 slices whole wheat toast w/1 Tbsp. Preserves and ½ grapefruit
3. Consume 8-10 servings of fruits and vegetables each day.
4. Consume 2-3 servings of low fat dairy daily. This may include:
   1. 1 oz of low-fat cheese
   2. 1 cup skim milk or 1% milk
   3. 1 cup fat free or low-fat yogurt
5. Consume a small portion of nuts, seeds, or legumes 4-5 days per week approximately equal to 20 low-sodium nuts, 2 tbsp. seeds, and ½ cup cooked dried beans.
6. Limit alcohol consumption to no more than 1 drink per day for women and 2 drinks per day for men.
7. Choose at least 6 Servings of whole grains per day (e.g. whole wheat bread, brown rice, whole wheat pasta, and other whole grain products)
8. Consume 2 servings of healthy lean protein per day such as 3 ounces portions of low fat meats, skinless chicken, or fish.
9. Eliminate high fat snacks

### Physical Activity / Sedentary Behavior Goals

1. Brisk walking for at least 20 minutes, 6 days per week, preferably in bouts ≥ 10 mins. Alternatively, choose one of the following activities as a replacement
   - 1. 20 minutes of bicycling about 5-9 miles per hour using a stationary bike or on flat roads if bicycling outside
     2. 20 minutes of recreational swimming or water aerobics
     3. 20 minutes dancing – high intensity
     4. 20 minutes of using a stair climber machine at a light-moderate pace
     5. 20 minutes of playing tennis
2. Reach 10,000 steps each day, increasing in increments of 2500 steps until the 10k steps/d goal is reached.
3. Limit television watching to less than 2 hours per day.
4. Perform strength training exercises at least 2 days per week

**Behavioral Goals**

1. Daily self-monitoring of weight (at the same time each day)
2. Exercise before the day begins (e.g. work, school, etc.)]
3. Avoid late-night eating after dinner (i.e. after 8pm if you eat dinner around 6pm).
4. Avoid f*ast food r*estaurants. If unable to avoid these restaurants, choose healthier options such as a salad with fat-free or no dressing or a fruit cup.

**Literature cited**

Bennett GG, Wolin KY, Puleo E, Emmons KM (2006a) Pedometer-determined physical activity among multiethnic low-income housing residents. *Med Sci Sports Exerc* **38:** 768-73

Bennett GG, Wolin KY, Viswanath K, Askew S, Puleo E, Emmons KM (2006b) Television viewing and pedometer-determined physical activity among multiethnic residents of low-income housing. *Am J Public Health* **96:** 1681-5

Berkey CS, Rockett HR, Gillman MW, Colditz GA (2003) One-year changes in activity and in inactivity among 10- to 15-year-old boys and girls: relationship to change in body mass index. *Pediatrics* **111:** 836-43.

Diabetes Prevention Program Research Group (2002) Reduction in the Incidence of Type 2 Diabetes with Lifestyle Intervention or Metformin. *N Engl J Med* **346:** 393-403

Glasgow RE, Bull SS, Piette JD, Steiner JF (2004) Interactive behavior change technology. A partial solution to the competing demands of primary care. *Am J Prev Med* **27:** 80-7

Hu FB, Li TY, Colditz GA, Willett WC, Manson JE (2003) Television watching and other sedentary behaviors in relation to risk of obesity and type 2 diabetes mellitus in women. *Jama* **289:** 1785-91

NHLBI Obesity Education Initiative Expert panel on the Identification E, and Treatment of Overweight and Obesity in Adults, (1998) *Clinical Guidelines on the Identification, Evaluation, and Treatment of Overweight and Obesity in Adults*edn. Bethesda, Maryland: National Heart, Lung, and Blood Institute, National Institutes of Health

Sallis J, Owen N (1999) *Physical activity and behavioral medicine*. Vol. 3edn. Thousand Oaks, CA: SAGE Publications Ltd

Sallis JF, Bauman A, Pratt M (1998) Environmental and policy interventions to promote physical activity. *Am J Prev Med* **15:** 379-97
